# Assessment of deep learning reconstruction effects on detection and differentiation of liver metastasis from hepatic hemangioma in diffusion-weighted imaging

**DOI:** 10.1007/s11604-025-01904-4

**Published:** 2025-11-06

**Authors:** Kumi Ozaki, Hanae Hasegawa, Shota Ishida, Jihun Kwon, Yasutomo Katsumata, Masami Yoneyama, Yukichi Tanahashi, Satoshi Goshima

**Affiliations:** 1 Department of Radiology, Hamamatsu University School of Medicine, 1-20-1, Handayama, Chuo-ku, Hamamatsu City, Shizuoka 431-3192 Japan; 2 Department of Radiological Technology, Faculty of Medical Science, Kyoto College of Medical Science, Nantan City, Kyoto Japan; 3 Philips Japan, Azabudai Hills Mori JP Tower 15F, 1-3-1 Azabudai, Minato-ku, Tokyo, 106-0041 Japan

**Keywords:** Diffusion weighted imaging, Apparent diffusion coefficient, Deep learning reconstruction, Liver, Liver metastasis, Hepatic hemangioma

## Abstract

**Purpose:**

To evaluate and compare the performance of diffusion-weighted imaging (DWI) using compressed sensing (CS) and DWI using CS with model-based deep learning reconstruction (DL-DWI) in detecting and differentiating liver metastases from hepatic hemangiomas.

**Materials and methods:**

We retrospectively analyzed data from 53 patients with metastases or hemangiomas (34 men and 19 women, mean age, 65.9 years) who underwent abdominal DWI. Two radiologists evaluated liver contour and distortion, artifact, noise, overall image quality, and lesion conspicuity using a five-point scale. Signal-to-noise ratio (SNR) and apparent diffusion coefficient (ADC) of the liver, as well as contras-to-noise ratio (CNR) and ADC of metastases (n = 59) and hemangiomas (n = 33) were assessed and statistically compared. A receiver operating characteristic (ROC) analysis was performed to assess the diagnostic performance of the two sequences for differentiating metastases and hemangiomas.

**Results:**

DL-DWI provided significantly better conspicuity of metastasis than CS-DWI (p < 0.05 in both radiologists), whereas no significant difference was observed in the conspicuity of hemangioma between DL-DWI and CS-DWI. The SNR of liver parenchyma and the CNR of metastases and hemangiomas were higher in DL-DWI than in CS-DWI (p < 0.05). ADC values of liver parenchyma, metastases, and hemangiomas were lower in DL-DWI than in CS-DWI (p < 0.05). The ADC cutoff value for differentiating between metastases and hemangiomas was 1.693 × 10^–3^ mm^2^/s in DL-DWI and 1.411 × 10^–3^ mm^2^/s in CS-DWI. No significant differences were observed in the area under the ROC curve, sensitivity, and specificity between the two methods (p > 0.05).

**Conclusion:**

DL-DWI enhanced both qualitative and quantitative aspects of image quality in abdominal DWI. However, its diagnostic performance, including ADC cutoff values for differentiating between metastases and hemangiomas, is comparable to that of CS-DWI.

## Introduction

Diffusion-weighted imaging (DWI) visualizes tissue characteristics by measuring the random motion of water molecules, thereby providing valuable information about the tissue microenvironment, such as tissue cellularity and cell membrane integrity [1]. DWI and its corresponding apparent diffusion coefficient (ADC) map have become essential sequences that significantly assist in lesion detection and diagnosis of various diseases, including inflammatory conditions and neoplasms of the upper abdomen [2, 3]. An important clinical application of upper abdominal DWI is the detection and characterization of liver metastases, which is crucial in staging and treatment planning, particularly for patients with colorectal cancer and other primary malignancies [4, 5]. The differential diagnosis of liver metastases from hemangiomas is clinically important, as T2-weighted imaging and dynamic contrast-enhanced studies often pose challenges in distinguishing these lesions [6, 7], making DWI a critical imaging tool for lesion differentiation.

The most commonly used pulse sequence for DWI is single-shot echo-planar imaging (EPI), which samples the entire k-space with a single excitation pulse, enabling rapid image acquisition [8]. This technique offers advantages including fast acquisition times and robustness against bulk motion, but it has inherent limitations such as low signal-to-noise ratio (SNR), reduced spatial resolution, and geometric distortion [9, 10]. Parallel imaging (PI) technology can reduce these distortions [11]; however, increasing the acceleration factor with PI decreases SNR, particularly in regions with high geometric (g-) factors such as the central portion of the image [12]. In addition, the high b-values recommended for the diagnosis of focal liver lesions often result in an insufficient SNR in DWI. Therefore, effective noise reduction methods are highly desirable to address these limitations.

In recent years, compressed sensing (CS) image acquisition and reconstruction techniques have been increasingly used in abdominal MRI [13, 14]. CS is an acceleration technique that requires random undersampling of k-space data, high sparsity of image signals, and nonlinear reconstruction. When combined with PI, CS reconstruction employs iterative L1-regularized denoising filters, such as wavelet-based approaches, to achieve an optimal balance between noise reduction and data consistency [15, 16]. Furthermore, previous studies have shown that applying the CS framework to EPI can reduce noise-like artifacts and improve image quality of EPI-based DWI without further optimization of the EPI sampling scheme [17, 18].

Recent developments in deep learning (DL) with convolutional neural networks (CNNs) have significantly advanced medical imaging applications [19, 20], particularly DL-based image reconstruction techniques, which have demonstrated promising results in denoising for MRI data acquisition and reconstruction [21, 22]. Notably, model-based DL reconstruction techniques represent a cutting-edge development in denoising magnetic resonance images, providing significant advancements in the field [23, 24].

This emergent model-based DL technique holds significant potential for effective noise removal in DWI and is expected to provide superior denoising performance in challenging imaging scenarios across various anatomical regions and pathological conditions [24–26]. However, its clinical utility for detecting and differentiating hepatic lesions has not yet been validated.

Therefore, we aimed to evaluate and compare the performance of diffusion-weighted imaging (DWI) using compressed sensing (CS) and DWI using compressed sensing with model-based deep learning reconstruction (DL-DWI) in detecting and differentiating liver metastases from hepatic hemangiomasDL-DWI.

## Materials and methods

The Research Ethics Committee of our institution approved this study (Approval No. 24–159) and waived the requirement for written informed consent due to the retrospective nature of this study.

### Patients

**Fig. 1:**
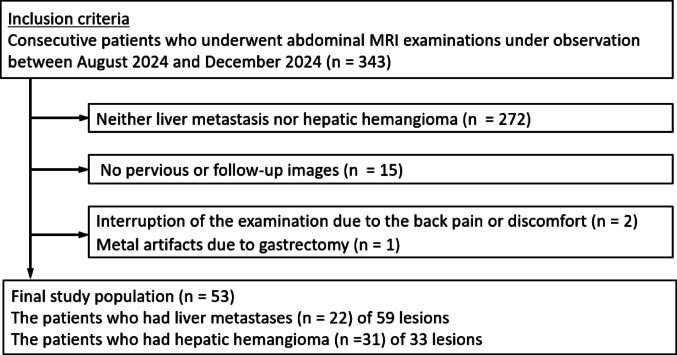
Flowchart of patient selection

We identified 343 consecutive patients who underwent abdominal 3-T magnetic resonance imaging (MRI) examinations between August and December 2024. Patients with liver metastasis or hepatic hemangioma, and prior or follow-up computed tomography (CT) or MR images spanning at least 6 months or more were enrolled in this study. We excluded those whose examination was interrupted (n = 2) and those with metal artifacts due to gastrectomy (n = 1). The demographics of the study population are presented in Fig. 1.

### MR examinations

MR examinations were performed using a 3 T MRI unit (MR7700; Philips Healthcare, Best, the Netherlands) with a 32-channel ds Torso coil. DWI was obtained using fat-suppressed two-dimensional single-shot EPI with CS reconstruction at b values of 0 and 800 s/mm^2^. Table 1 shows the details of the imaging parameters and the acquisition time. ADC maps were calculated automatically in the MR console using b-values of 0 and 800 s/mm^2^ with a mono-exponential model.

**Table 1:** Acquisition parameters of diffusion-weighted images and other sequences

	Diffusion weighted imaging	Chemical shift imaging	T2-weighted imaging	Single-shot turbo spin echo	Dynamic contrast study	Hepatobiliary phase
Sequence	EPI	FFE	Motion free	Turbo spin echo	e-THRIVE	e-THRIVE
Fat suppression	STIR	-	SPIR	SPAIR	SPAIR	SPAIR
Acquisition plane	Transverse	Transverse	Transverse	Transverse	Transverse	Transverse
Respiratory compensation	Respiratory trigger	Breath-hold	Respiratory trigger	Breath-hold	Breath-hold	Breath-hold
Repetition/echo time (ms)	Range (1000–1400)/ 64	200/2.3 or 1.15	Range 1200–1700/77	Shortest (5697)/82	3/1.5	3/1.5
Echo train		-	28	-	-	-
Flip angle (degrees)	90	60	90	90	10	15
Section thickness/gap (mm)	5.0/1.0	5.0/1.0	5.0/1.0	5.0/1.0	4.0/0	4.0/0
Number of slices	34	34	34	36	100	100
Field of view (mm^2^)	360 × 304	360 × 280	360 × 284	360 × 298	360 × 300	360 × 300
Acquisition voxel (mm^3^)	2.81 × 2.83 × 5.0	1.61 × 1.50 × 5.00	1.34 × 1.34 × 5.0	1.30 × 1.79 × 5.0	1.2 × 1.5 × 3.4	1.3 × 1.6 × 3.4
Recon matrix	256	448	512	512	512	512
Recon voxel (mm^3^)	1.41 × 1.41 × 5.0	0.8 × 0.8 × 5.0	0.7 × 0.7 × 5.0	0.7 × 0.7 × 5.0	0.7 × 0.7 × 2.0	0.7 × 0.7 × 1.7
BW in EPI freq dir (Hz)	2742.7	1535.9	545.5	503.2	868.5	722.7
Parallel imaging (PI)	CS-SENSE/AI CS-SENSE	AI CS-SENSE	AI CS-SENSE	AI CS-SENSE	AI CS-SENSE	CS-SENSE
PI factor	2.5	2.4	2.5	4.5	4.5	3.7
Half scan	0.892	0.85	No	0.9	No	No
Matrix	128 × 107	224 × 193	268 × 211	276 × 167	320 × 192	320 × 224
NSA	b0 (2), b 800 (3)	1	1.4	1	1	1
Acquisition time (sec)	144	16.3	164	13.5	13.1 × 4	16

*Dynamic contrast study consists of pre-contrast, arterial, portal venous, and transitional phases. In dynamic contrast study, a dose of 0.1 mL/kg of gadoxetic acid was administered at a rate of 1 mL/s followed by a 20-mL saline flush using a power injector and was used a bolus tracking technique

EPI: echo-planar imaging, FFE: fast field echo, SPIR; spectral presaturation with inversion recovery, e-THRIVE; T1-high resolution isotropic volume excitation, STIR: short tau inversion recovery. SPIR = spectral presaturation with inversion recovery, SPAIR = spectral attenuated inversion recovery, NEX: number of excitations, CS-SENSE = compressed SENSE, AI CS-SENSE = compressed SENSE with deep learning reconstruction, NSA: number of sample [signals] averaged

### Model-based DL reconstruction

We implemented a model-based DL reconstruction technique using Adaptive-CS-Net, deployed through a vendor prototype (Next Generation Scan Acceleration patch). The comprehensive training and optimization methods for Adaptive-CS-Net have been previously described [23]. This model-based DL reconstruction technique achieves effective denoising by performing iterative image processing on undersampled k-space data using the Adaptive-CS-Net architecture. In this model’s pipeline, Adaptive-CS-Net serves as a functional replacement for the wavelet transform component in conventional CS-based image reconstruction algorithms. The network architecture comprises a U-Net structure integrated with a soft thresholding function, enabling effective image denoising through sparsification.

A distinctive feature of this reconstruction model is its integration of domain-specific prior knowledge for verifying data consistency. At each iteration, the system performs consistency checks by comparing images before and after processing through Adaptive-CS-Net to ensure optimal denoising performance. This iterative framework facilitates the removal of noise while preserving and enhancing the original signal, ultimately achieving substantial improvements in image quality. All image processing was performed within the MR system’s console.

In this study, we used an enhanced version of Adaptive-CS-Net beyond that originally described in Pezzotti et al. [23], and incorporated additional improvements detailed in Foreman et al. [24]. The enhanced network underwent extensive pretraining on a large, diverse dataset comprising 1.5T and 3T images across multiple anatomical regions and contrast mechanisms. The network was optimized for execution on standard reconstruction hardware to ensure a robust performance and broad clinical applicability. Previous clinical evaluations have demonstrated the effectiveness of this approach for head and neck and prostate DWI applications [25, 26].

We intentionally excluded PI-based acceleration, commonly employed in similar studies to isolate the image quality improvement effects of model-based DL reconstruction. This methodological approach enabled single DWI acquisitions rather than comparative dual scans, thereby eliminating potential confounding factors such as variations in acquisition conditions and incidental patient movement between consecutive scans. Consequently, the reconstruction was performed on the console using DWI using single-shot EPI with CS (CS-DWI), yielding the following four image types: CS-DWI, CS-DWI with model-based DL reconstruction-enhanced CS (DL-DWI), and ADC maps based on CS-DWI and DL-DWI.

### Identification of hepatic lesions and imaging diagnosis

Two board-certified radiologists (with 25 and 29 years of post-training experience in interpreting abdominal MRI images) detected and diagnosed all liver metastases and hepatic hemangiomas in consensus with reference to all MRI sequences obtained simultaneously and previous and following contrast-enhanced CT and MR images spanning at least 6 months or more.

Hemangiomas were diagnosed based on characteristic imaging findings, including nodular or peripheral discontinuous enhancement during the arterial phase and progressive centripetal fill-in enhancement during the portal venous or delayed phases on contrast-enhanced CT or MRI. On T2-weighted and fat-suppressed T2-weighted images, the lesions demonstrated hyperintensity relative to the liver parenchyma, but with signal intensity lower than that of water [27]. The diagnosis was further supported by the absence of malignancy in the patient’s medical history and documented lesion stability (no size change) over a minimum follow-up period of 6 months.

In contrast, liver metastases were diagnosed based on clinical history of pathologically confirmed malignancy combined with characteristic imaging findings. The diagnostic imaging criteria included peripheral and peritumoral enhancement during the arterial or portal venous phases on contrast-enhanced CT or MRI, and/or hypointensity on hepatobiliary phase images of gadoxetic acid-enhanced MRI [28]. Lesion appearance or size changes during the follow-up period provided additional diagnostic support. No hypervascular metastases were observed.

Two radiologists reported the diagnosis, size, and location of the lesions. All nodules detected and diagnosed by these two radiologists underwent qualitative evaluation by two additional radiologists as described below. Quantitative evaluation was performed for nodules measuring ≥ 5 mm. All image analyses were conducted with the final diagnosis blinded to the evaluating radiologists.

### Quantitative image analysis

Two board-certified radiologists (readers 1 and 2, with 5 and 16 years of imaging experience, respectively) performed quantitative image analysis independently. Readers were blinded to sequence types, and could adjust the window settings. They were also blinded to the patient’s clinical information and the image acquisition method, and all images were anonymized. They reviewed two types of images presented independently in a random order. Although the qualitative diagnosis of the nodules was unknown (hemangioma or liver metastasis), the nodules to be measured were pre-specified. Hepatic cysts were not included in this study as they can be readily differentiated when assessed alongside other routine sequences, particularly T2-weighted imaging. All regions of interest (ROI) were drawn using the SAI viewer (SYNAPSE®, FUJIFILM Medical, Tokyo, Japan). Circular ROIs were placed in artifact-free, homogeneous areas (excluding vessels) on the right robe of the liver and on focal liver lesions to measure the signal intensity (SI), standard deviation (SD), and mean ADC values on DWI (*b* value = 800 s/mm^2^). Once an ROI was placed on one image, it was copied and pasted onto the other DWI image. Measurements were performed in triplicates to ensure data consistency and reproducibility, and the mean of the three ROIs were used to represent each region.

Given the geometric variation in noise across the field of view of the image resulting from the use of PI, the SD of SIs in the background air could not be used as a measurement of noise. Therefore, we calculated the estimated SNR of each object using the method proposed by Heverhagen, as follows [29]:

$$SNR = \frac{{SI_{liber} }}{{SD_{noise} }}$$

where SI_liver_ and SD_noise_ represent the mean SI and SD of the liver parenchyma and erector spinae, respectively.

The CNR for the focal liver lesions was calculated as follows [30]:

$${\mathrm{CNR}} = \left| {{\mathrm{SI}}_{{{\mathrm{liver}}}} - {\mathrm{SI}}_{{{\mathrm{lesion}}}} } \right|/\surd \left[ {\left( {{\mathrm{SD}}_{{{\mathrm{liver}}}}^{2} + {\mathrm{SD}}_{{{\mathrm{liver}}}}^{2} } \right)/2} \right]$$

where SI_liver_ and SD_liver_ represent the SI and SD of the liver parenchyma, and SI_lesion_ and SD_lesion_ represent the SI and SD of the focal liver lesion.

### Qualitative image analysis

The same board-certified radiologists independently evaluated the CS-DWI and DL-DWI at a *b* value of 800 s/mm^2^. The evaluations included [31]: contour and distortion of the liver, artifacts (including motion, ringing, partial volume, and susceptibility artifacts), and overall image quality (as reflected in the general image impression). Each evaluation was scored using a 5-point Likert scale, and the conspicuity of the hepatic lesions was also assessed. The criteria are listed in Table 2.

**Table 2:** Scoring criteria for qualitative image evaluation

Score	Contour of the liver	Distortion of the liver	Image noise	Artifacts	Overall image quality	Lesion conspicuity
5	Smooth regular contour	Normal liver morphology	No noise	No artifacts	Excellent	Complete lesion visualization, optimal for detection
4	Mild surface nodularity	Minimal surface irregularity	Minimal noise	Minimal artifacts	Good	Good lesion visualization, adequate for detection
3	Moderate undulating border	Moderate contour deformation	Mild noise causing little impact on diagnosis	Mild artifacts causing little impact on diagnosis	Acceptable	Acceptable lesion visualization, detection possible
2	Irregular coarse margin	Marked architectural distortion	Moderate noise causing substantial impact	Moderate artifacts causing substantial impact on diagnosis	Moderately poor	Limited lesion visualization, detection difficult
1	Severely distorted outline	Severe structural derangement	Nondiagnostic due to severe noise	Severe artifacts	Definitely poor	Poor lesion visualization, detection not feasible

### Statistical analysis

Continuous variables are expressed as mean ± SD. Data normality was confirmed using the Shapiro–Wilk test. Subsequently, SNR of the liver parenchyma, CNR of the lesions, and mean ADC values of the liver parenchyma and lesions were compared between the CS-DWI and DL-DWI using the Wilcoxon signed-rank test. Intra-observer agreement of each reader and interobserver agreement between the two readers for SNR and CNR measurements were evaluated using intraclass correlation coefficients (ICCs). A 2-way mixed model was employed to calculate ICCs with 95% confidence intervals. ICC (3,1) was used to assess intra-observer agreement within each reader’s three repeated measurements, and ICC (2,1) was used to evaluate inter-observer agreement between the two readers. Agreement based on the ICCs was classified using the following definitions: < 0.50, poor; 0.50–0.74, moderate; 0.75–0.89, good; and 0.90–1.00, excellent [32]. Qualitative image scores were compared between the CS-DWI and DL-DWI using the Wilcoxon signed-rank test. Cohen’s weighted Kappa analysis assessed interreader agreement (0.01–0.20, poor; 0.21–0.40, fair; 0.41–0.60, moderate; 0.61–0.80, substantial; and 0.81–1.00, almost perfect agreements) [33].

The optimal ADC cutoff value for differentiating liver metastases from hemangiomas was determined using receiver operating characteristic (ROC) analysis, which yielded the maximal sensitivity and specificity. The diagnostic performance of CS-DWI and DL-DWI was evaluated using the optimal ADC cutoff values. In ROC analyses, the mean ADC values reported by the two radiologists were considered representative of the ADC values. The area under the ROC curve (AUC) was compared between CS-DWI and DL-DWI using DeLong’s test. The sensitivity and specificity for differentiating between the two entities in CS-DWI and DL-DWI were examined using McNemar’s test. For all statistical analyses, two-sided p-values of < 0.05 denoted statistical significance. All statistical analyses were performed using SPSS software (version 27.0; IBM Corp., Armonk, NY, USA).

## Results

### Clinical characteristics of patients

**Fig. 2:**
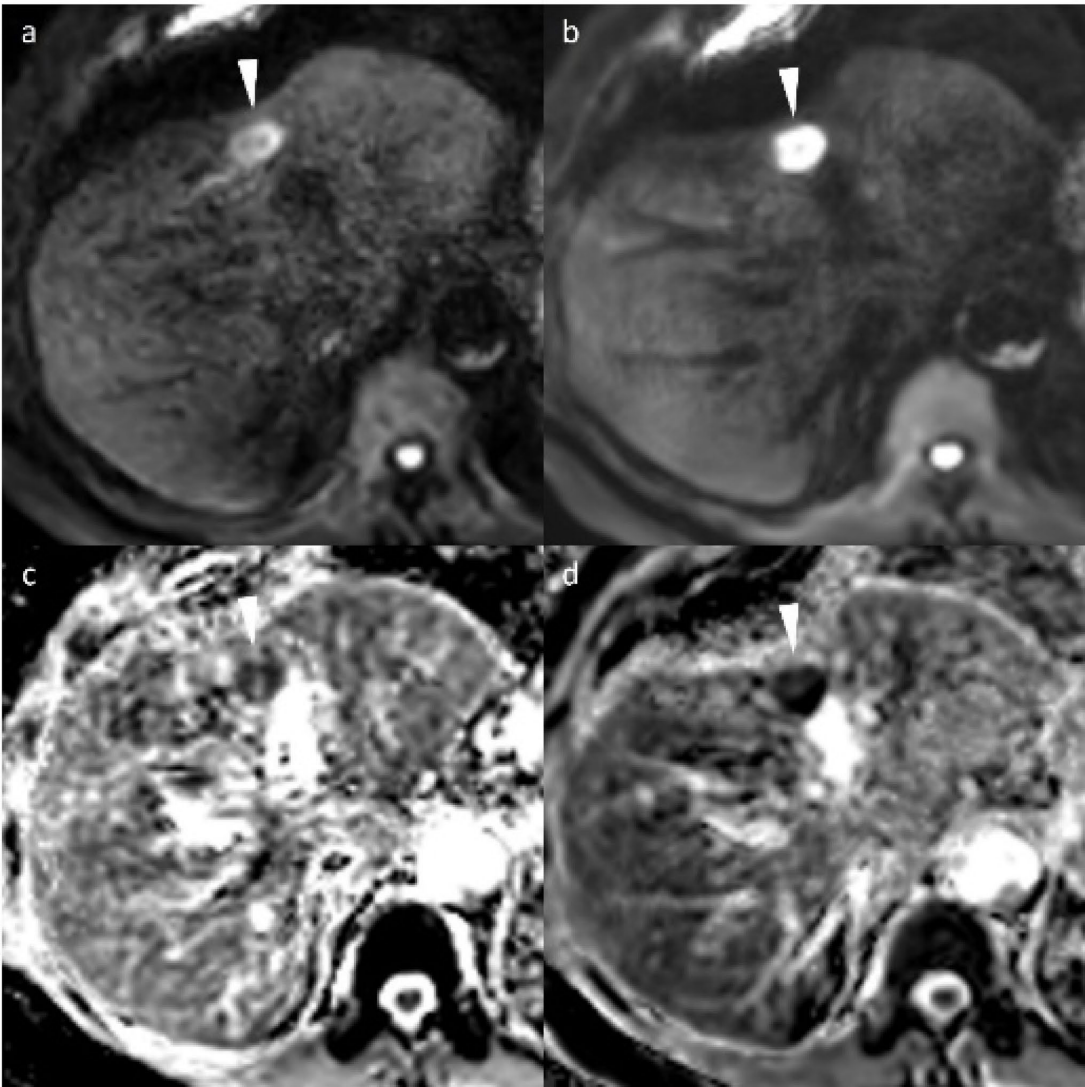
Representative cases of liver metastasis in a 78-year-old man with sigmoid colon cancer. **a** DWI (b-value = 800 s/mm^2^) image with compressed sensing (CS-DWI). **b** DWI image with CS and model-based image reconstruction (DL-DWI). **c** ADC map (derived from b0 and b800) of CS-DWI. (d) ADC map (derived from b0 and b800) of DL-DWI. A 16-mm liver metastasis in segment 4 demonstrated high signal intensity on DWI; however, it appeared distorted with decreased signal attenuation due to artifacts on CS-DWI (**a**). DL-DWI (**b**) provides superior visualization of the entire nodule. The ADC map of CS-DWI (**c**) shows more prominent distortion and artifacts compared to that of DL-DWI (**d**). DWI = diffusion-weighted imaging, CS-DWI = DWI using single-shot echo planar imaging with compressed sensing, DL-DWI = DWI using echo planar imaging with model-based deep learning (DL) reconstruction-enhanced CS, ADC = apparent diffusion coefficient

**Fig. 3:**
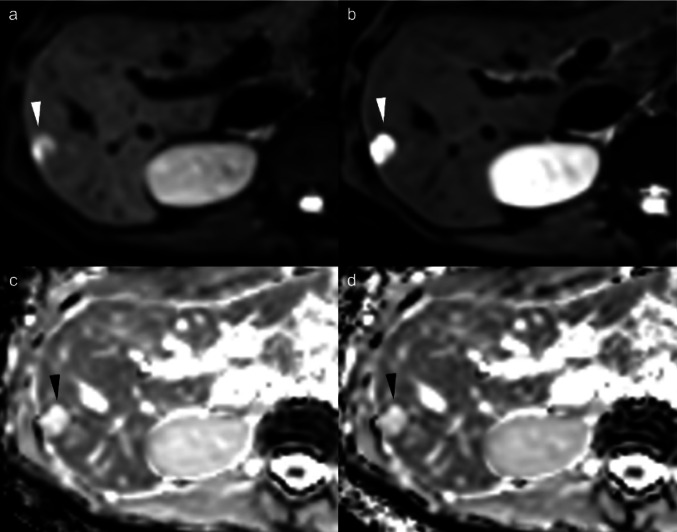
Representative cases of hepatic hemangioma in a 47-year-old woman. **a** DWI (b-value = 800 s/mm^2^) image with compressed sensing (CS-DWI). **b** DWI image with CS and model-based image reconstruction (DL-DWI). **c** ADC map (derived from b0 and b800) of CS-DWI. **d** ADC map (derived from b0 and b800) of DL-DWI. A 32-mm hepatic metastasis in segment 6 demonstrated high signal intensity on DWI; however, it appeared distorted with decreased signal attenuation due to artifacts on CS-DWI (**a**). DL-DWI (**b**) provides superior visualization of the entire nodule. DWI = diffusion-weighted imaging, CS-DWI = DWI using single-shot echo planar imaging with compressed sensing, DL-DWI = DWI using echo planar imaging with model-based deep learning (DL) reconstruction-enhanced CS, ADC = apparent diffusion coefficient

This study included 53 consecutive patients (22 with liver metastases and 31 with hepatic hemangiomas): 23 males and 38 females with a mean age of 65.9 ± 10.1 years. None of the patients had both lesions. Those with metastases were significantly older than those with hemangiomas (p < 0.05). Metastases were significantly larger than hemangiomas (p < 0.05). Additional details on patients and lesions characteristics are presented in Table 3. Representative cases are shown in Figs. 2 and 3.

**Table 3:** Characteristics of the subjects

Characteristics	Total	Liver metastasis	Hepatic hemangioma	P value
Case	53	22	31	–
Age, years	65.9 ± 10.1	70.6 ± 7.4	63.1 ± 10.7	0.003
Sex; male/female	34/19	12/10	22/9	0.284
Body weight	61.0 ± 13.0	58.3 ± 12.8	62.9 ± 13.0	0.889
Hight	162.2 ± 8.3	160.1 ± 9.9	163.5 ± 6.9	0.153
Body surface area	1.64 ± 0.19	1.60 ± 0.04	1.68 ± 0.19	0.802
Nodules				
Number of lesions	137	78	59	–
Number of lesions (1/≧ 2)	44/9	15/7	29/2	–
≧ 5 mm/ < 5 mm	92/45	59/19	22/26	–
Size (mm)	12.1 ± 8.5	14.2 ± 8.7	8.9 ± 8.5	< 0.001
Location (lateral, medial, anterior, and posterior segments, and caudate lobe)	17/13/26/31/5	10/9/20/18/2	7/4/6/13/3	0.307
Lesions within 1 cm of the liver surface	51	28	23	0.199
Primary tumor	–	Colorectal (10), pancreatic (5), gastric cancer (3), and others (4)	–	–

Continuous variables are expressed as mean ± standard deviation. Categorical variables are expressed as the number of cases

### Quantitative image evaluation

**Fig. 4:**
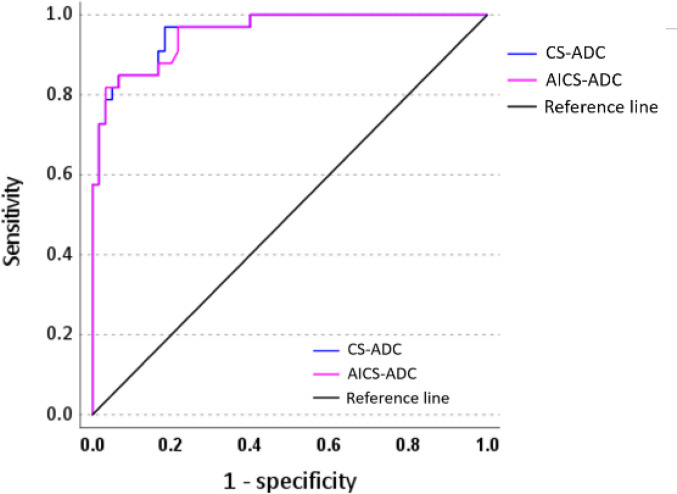
Receiver operating characteristic curves for differentiating liver metastases from hepatic hemangiomas by ADC values. No significant differences were observed in AUC, sensitivity, or specificity between CS-DWI and DL-DWI in differentiating liver metastases from hemangiomas based on ADC values. DWI = diffusion-weighted imaging, CS-DWI = DWI using single-shot echo planar imaging with compressed sensing, DL-DWI = DWI using echo planar imaging with model-based deep learning (DL) reconstruction-enhanced CS, ADC = apparent diffusion coefficient, AUC = area under the receiver operating characteristic curve

The SNR of liver parenchyma in DL-DWI was significantly higher, while the ADC values were significantly lower than those in CS-DWI (p < 0.05). The CNR and ADC values of liver metastases and hepatic hemangiomas in DL-DWI were significantly higher than those in CS-DWI (p < 0.05). Good to excellent intra-observer agreement was found for both readers’ measurements (ICC [1, 3] = 0.907–0.950 and 0.920–0.981, respectively). Good agreement was found between the two readers’ measurements (ICC [1, 2] = 0.751–0.852) (Table 4). No significant difference was observed between CS-DWI and DL-DWI in AUC (0.959 and 0.956 in CS-DWI and DL-DWI, respectively; p = 0.853), sensitivity (81.7% and 81.8% in CS-DWI and DL-DWI, respectively; p > 0.999), and specificity (97.0% and 96.7% in CS-DWI and DL-DWI, respectively; p > 0.999; Fig. 4). To differentiate liver metastases from hepatic hemangiomas, the optimal cutoff ADC values were 1.411 × 10^–6^ mm^2^/s and 1.693 × 10^–6^ mm^2^/s for CS-DWI and DL-DWI respectively.

**Table 4:** Quantitative image analyses results

	CS-DWI	DL-DWI	P value	ICC (2, 1) [95% CI]
*Liver parenchyma (n = 53)*				
*SNR*				0.842 [0.792–0.889]
Reader 1	9.71 ± 4.67	11.00 ± 5.63	< 0.001	
ICC (3, 1) [95% CI]	0.936 [0.922–0.955]	0.931 [0.919–0.950]	–	
Reader 2	13.88 ± 6.53	17.12 ± 9.80	< 0.001	
ICC (3, 1) [95% CI]	0.969 [0.959–0.978]	0.967 [0.958–0.974]	–	
*ADC value (× 10*^*–6*^* mm*^*2*^*/s)*				0.849 [0.791–0.888]
Reader 1	1.370 ± 0.245	1.322 ± 0.317	< 0.001	
ICC (3, 1) [95% CI]	0.907 [0.901–0.916]	0.911 [0.903–0.917]	–	
Reader 2	1.258 ± 0.209	1.169 ± 0.188	< 0.001	
ICC (3, 1) [95% CI]	0.927 [0.915–0.936]	0.920 [0.914–0.928]	–	
*Liver metastases (n = 59)*				
*CNR*				0.811 [0.765–0.849]
Reader 1	25.6 ± 16.6	28.4 ± 18.4	< 0.001	
ICC (3, 1) [95% CI]	0.950 [0.938–0.961]	0.946 [0.932–0.955]	–	
Reader 2	42.4 ± 26.7	48.9 ± 30.1	< 0.001	
ICC (3, 1) [95% CI]	0.971 [0.958–0.983]	0.977 [0.960–0.988]	–	
*ADC value (× 10*^*–6*^* mm*^*2*^*/s)*				0.751 [0.701–0.823]
Reader 1	1.042 ± 0.407	1.010 ± 0.421	< 0.001	
ICC (3, 1) [95% CI]	0.911 [0.903–0.920]	0.921 [0.913–0.933]		
Reader 2	1.105 ± 0.491	1.079 ± 0.510	< 0.001	
ICC (3, 1) [95% CI]	0.961 [0.943–0.968]	0.963 [0.965–0.989]	–	
*Hepatic hemangioma (n = 33)*				
*CNR*				0.852 [0.735–0.915]
Reader 1	22.58 ± 18.93	27.09 ± 22.37	< 0.001	
ICC (3, 1) [95% CI]	0.925 [0.920–0.933]	0.929 [0.921–0.938]		
Reader 2	23.03 ± 16.69	28.20 ± 21.49	< 0.001	
ICC (3, 1) [95% CI]	0.975 [0.961–0.985]	0.981 [0.965–0.989]	–	
*ADC value (× 10*^*–6*^* mm*^*2*^*/s)*				0.793 [0.660–0.878]
Reader 1	1.977 ± 0.466	2.000 ± 0.479	< 0.001	
ICC (3, 1) [95% CI]	0.920 [0.902–0.941]	0.932 [0.914–0.974]		
Reader 2	1.792 ± 0.554	1.776 ± 0.545	< 0.001	
ICC (3, 1) [95% CI]	0.951 [0.938–0.963]	0.950 [0.940–0.961]	–	

Data are expressed as mean score ± standard deviation

CS-DWI, diffusion-weighted imaging (DWI) using single-shot echo planar imaging with compressed sensing (CS); DL-DWI, DWI using echo planar imaging with model-based deep learning (DL) reconstruction-enhanced CS; ICC, intraclass correlation coefficient; CI, confidence interval; SNR, signal-to-noise ratio; ADC, apparent diffusion coefficient; CNR, contrast-to-noise ratio

### Qualitative image analysis

**Fig. 5:**
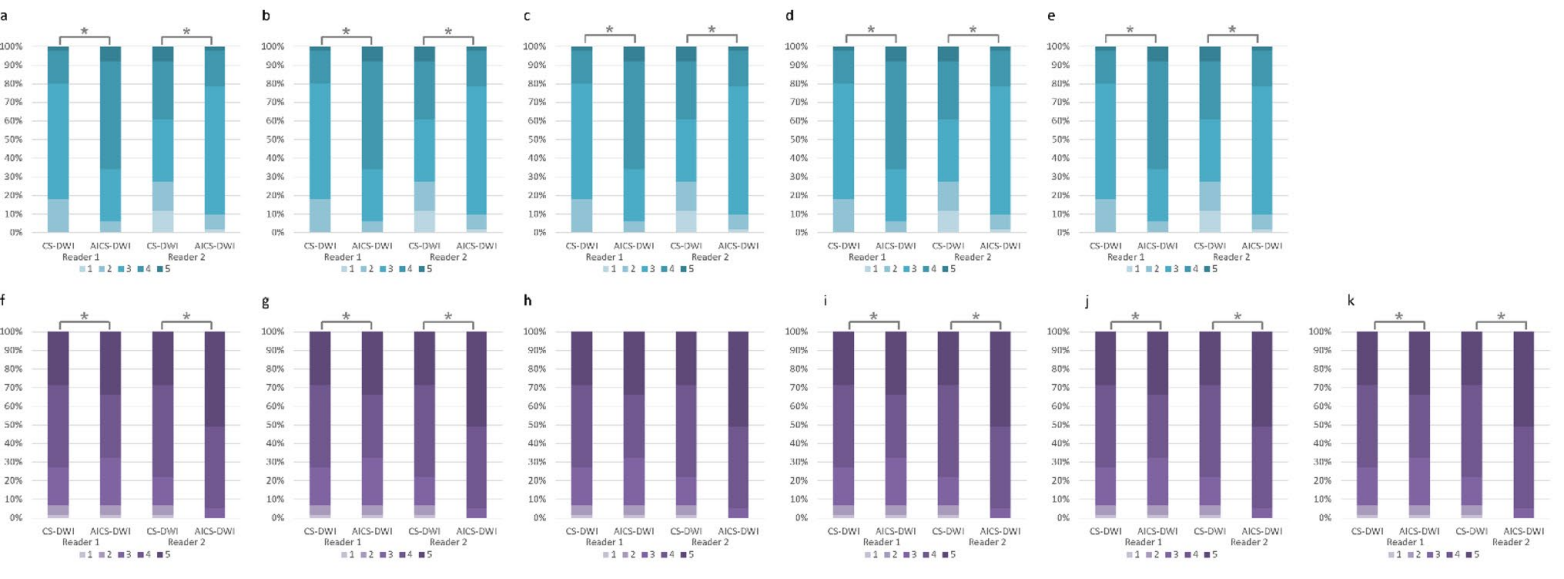
Stacked bar charts for qualitative image assessment stratified. a. Distortion of the liver, b. Liver contour, c. Artifact, d. Image noise, e. Overall image quality, f. All lesion conspicuity, g. Conspicuity of liver metastasis, h. Conspicuity of hepatic hemangioma, i. Lesion conspicuity within 1 cm from the liver surface, j. Liver metastasis conspicuity within 1 cm from the liver surface, k. Hepatic hemangioma conspicuity within 1 cm from the liver surface. An asterisk (*) indicates a significant difference (p < 0.05)

DL-DWI showed significantly high image qualities regarding liver contour and distortion, image noise, artifact, and overall image quality compared with CS-DWI (p < 0.05). Concerning the lesion conspicuity, no significant differences were observed in hepatic hemangioma conspicuity between DL-DWI and CS-DWI in Reader 1 (p = 0.028). In others, the conspicuity of lesions on DL-DWI images was significantly higher than that on CS-DWI images (p < 0.05). Particularly, lesions within 1 cm from the liver surface showed significantly higher conspicuity on DL-DWI compared to CS-DWI for Readers 1 and 2 (p < 0.05). The results showed fair to moderate agreement (Cohen’s κ; 0.58–0.69) (Table 5 and Fig. 5).

**Table 5:** Qualitative image analyses results

	CS-DWI	DL-DWI	P value	κ-value
*Distortion of the liver*				0.67
Reader 1	3 (3–4)	4 (3–4)	< 0.001	
Reader 2	3 (2–4)	4 (4–4)	< 0.001	
*Liver contour*				0.59
Reader 1	4 (3–5)	4 (4–5)	0.005	
Reader 2	3 (3–4)	4 (4–4)	< 0.001	
*Artifact*				0.68
Reader 1	3 (3–4)	4 (3–4)	0.001	
Reader 2	3 (3–3)	4 (3–4)	< 0.001	
*Image noise*				0.61
Reader 1	3 (2–4)	4 (3–5)	0.001	
Reader 2	3 (3–4)	4 (4–4)	< 0.001	
*Overall image quality*				0.69
Reader 1	3 (3–4)	4 (3–5)	< 0.001	
Reader 2	3 (3–4)	4 (4–4)	< 0.001	
*Lesion conspicuity of all lesions (n = 137*)				0.63
Reader 1	4 (3–5)	4 (4–5)	0.028	
Reader 2	5 (3–5)	5 (4–5)	< 0.001	
*Liver metastasis (n = 78)*				0.62
Reader 1	5 (3–5)	5 (4–5)	0.028	
Reader 2	5 (3.25–5)	5 (4–5)	0.002	
*Hepatic hemangioma (n = 59)*				0.63
Reader 1	4 (3–5)	4 (3–5)	0.637	
Reader 2	4 (3–5)	4 (3–5)	0.382	
*Lesions within 1 cm from the liver surface (n = 51*)				0.62
Reader 1	4 (3–4)	5 (4–5)	0.028	
Reader 2	4 (3–4)	5 (4–5)	0.002	
*Liver metastasis (n = 28)*				0.62
Reader 1	4 (3–4)	5 (4–5)	0.019	
Reader 2	4 (3–4)	5 (4–5)	0.002	
*Hepatic hemangioma (n = 23)*				0.62
Reader 1	4 (3–5)	5 (4–5)	0.003	
Reader 2	4 (3–4)	5 (4–5)	0.035	

Data are expressed as medians with interquartile range in parentheses

CS-DWI, diffusion-weighted imaging (DWI) using single-shot echo planar imaging with compressed sensing (CS); DL-DWI, DWI using echo planar imaging with model-based deep learning (DL) reconstruction-enhanced CS

## Discussion

This study demonstrated that DL-DWI significantly improved image quality and enhanced the conspicuity of hepatic lesions, including hemangiomas and liver metastases, compared to conventional CS-DWI, particularly for nodules located within 1 cm of the liver surface. These findings represent a significant advancement in abdominal DWI technology with important clinical implications. Our method of comparing techniques within single acquisitions rather than using separate scans strengthens the validity of our findings by eliminating potential confounding factors, such as patient movement, physiological variations, and scanner stability issues.

The superior performance of model-based DL reconstruction addresses the inherent limitations of conventional CS reconstruction. Single-shot EPI is the most frequently used technique in clinical DWI; however, combining it with PI methods to reduce geometric image distortions often decreases SNR in regions with high g-factors, particularly in the central area of the image. This reduction in SNR poses challenges in the evaluation of upper abdominal lesions. While the compressed SENSE framework applied to EPI (CS-DWI) reduces noise-like artifacts and improves image quality without further optimization of the EPI sampling scheme [17, 18], we speculate that noise and artifact reduction by L1 regularization might be limited, as it is an algorithm-based method with a set threshold value in the wavelet transformation process. The Adaptive-CS-Net architecture effectively addresses these limitations by leveraging learned features from large training datasets to achieve more advanced denoising than conventional wavelet-based approaches. The model-based DL reconstruction approach efficiently utilizes, processes, and integrates large amounts of signal data during iterative image processing, enabling effective noise reduction [23, 24] while preserving clinically relevant signal characteristics.

Our findings revealed that DL-DWI demonstrated significant improvements in lesion conspicuity as well as in liver contours, reduction of liver distortion, artifact, image noise, and overall image quality compared with CS-DWI. The scores for pancreatic distortion did not differ significantly, and this may be attributed to the fact that CS-DWI images had already achieved improved image quality through the compressed SENSE framework. The enhanced image quality resulted in improved lesion conspicuity for hemangiomas and liver metastases, particularly for lesions located within 1 cm from the liver surface. This selective improvement indicates enhanced image quality in regions susceptible to geometric distortion caused by B0 inhomogeneity, particularly areas in adjacent to air-containing structures, such as the lungs and regions prone to gastrointestinal artifacts. The lack of overall significant difference is likely due to the markedly high signal intensity of hemangiomas on b800 DWI due to T2-shine through effect, making them inherently conspicuous and rarely compromised to the extent that detection becomes challenging. The improvement in lesion visibility has direct clinical implications, potentially enhancing diagnostic confidence, reducing interpretation time, and facilitating earlier detection of pathological changes. The ability to better visualize hepatic lesions is particularly relevant in oncological applications where accurate lesion detection and characterization are critical for treatment planning and monitoring.

These quantitative improvements were further supported by the significantly higher SNR of the liver parenchyma and CNR of the focal liver lesions on DL-DWI than on CS-DWI. A crucial quantitative observation was that the ADC values of the liver and focal liver lesions on DL-DWI were significantly lower than those on CS-DWI images. Notably, some studies have reported no significant difference in ADC values between DL-DWI and conventional DWI in liver imaging [34], while others have reported lower ADC values in DL-DWI compared to conventional DWI in liver imaging [35]. However, these comparisons involved PI-DWI, and no studies have compared CS and AICS similar to our study. The underlying mechanisms remain unclear, although we speculate that the significant difference in background noise levels between DL-DWI and CS-DWI may have contributed to the observed differences in ADC value. Nevertheless, theoretically, ADC bias and random measurement error in DWI can be effectively reduced by model-based deep DL reconstruction [36].

The influence of model-based DL reconstruction on ADC values suggests that the cutoff ADC values used to differentiate between the two lesion types could be altered. Previous studies reported ADC cutoff values for differentiating liver metastases and hepatic hemangiomas ranging from 1.33 × 10⁻^3^ to 1.70 × 10⁻^3^ mm^2^/s [18, 37–39]. In our study, the ADC cutoff values of 1.693 × 10⁻^3^ mm^2^/s and 1.411 × 10⁻^3^ mm^2^/s measured on DL-DWI fell within this range. In addition, in this study, despite slight variations in the ADC cutoff values for distinguishing liver metastases from hepatic hemangiomas, no significant difference was observed in the discriminative ability between DL-DWI and CS-DWI. However, despite being within the reported range, the observed variations in cutoff values highlight that ADC measurements can be influenced by various factors, including equipment specifications, CS techniques, and different DL reconstruction methods. This variability underscores the critical need for continuous data updates as imaging equipment and reconstruction technologies evolve. In the ROC analysis using the cutoff values obtained in this study, the discriminative ability for differentiating liver metastases from hepatic hemangiomas was 0.956 DL-DWIand 0.959 for DL-DWI and CS-DWI, respectively, showing no significant difference between them. However, both values were higher than the AUC of 0.89 reported by Kaga et al. using CS-DWI analysis. We determined that ADC measurements could be obtained from images of nodules measuring ≥ 5 mm, which included smaller nodules compared to Kaga et al.'s study, which targeted nodules of ≥ 10 mm. We attribute these findings to the high specifications of the imaging equipment used in our study. Furthermore, establishing universally applicable diagnostic criteria that maintain reliability across different institutions would require extensive multi-center data collection and standardization efforts to account for these technical variations.

The present study had some limitations. First, the sample size was relatively small, and the study was conducted at a single center, which may have introduced selection bias. Therefore, our results should be regarded as preliminary. Second, MRI parameters such as acceleration factor, b-values, and acquisition time have not yet been optimized, requiring further trials for developing a standard methodology. Third, we did not directly compare our model-based DL reconstruction with other DL-based reconstruction techniques. Fourth, while we observed improved conspicuity of hemangiomas and liver metastases, quantitative assessment revealed no significant difference in the discriminative ability between hepatic metastases and hepatic hemangiomas. Further qualitative and quantitative investigations are needed for other types of focal liver lesions and lesions in other upper abdominal organs, such as gallbladder, pancreas, and spleen, to evaluate the feasibility of AICS, which may pose the risk of suppressing small anatomical details.

Future research should focus on a comprehensive evaluation of diagnostic performance for specific pathological conditions with histopathological correlation. Investigation across different b-values, optimization of acquisition parameters, and larger multi-center studies are needed to validate these preliminary findings and establish standardized protocols for clinical implementation.

In conclusion, this study provides compelling evidence that model-based DL reconstruction with Adaptive-CS-Net significantly enhances the clinical utility of abdominal DWI by improving image quality and lesion conspicuity, particularly for hemangiomas and liver metastases. These findings suggest that integrating advanced reconstruction techniques represent a promising direction for optimizing diffusion-weighted imaging in clinical practice, though further validation studies are required to establish its full clinical potential.

## References

[1] d’Assignies G, Fina P, Bruno O, Vullierme MP, Tubach F, Paradis V, et al. High sensitivity of diffusion-weighted MR imaging for the detection of liver metastases from neuroendocrine tumors: comparison with T2-weighted and dynamic gadolinium-enhanced MR imaging. Radiology. 2013;268:390–9. 10.1148/radiol.13121628. 23533288 10.1148/radiol.13121628

[2] Mottola JC, Sahni VA, Erturk SM, Swanson R, Banks PA, Mortele KJ. Diffusion-weighted MRI of focal cystic pancreatic lesions at 3.0-Tesla: preliminary results. Abdom Imaging. 2012;37:110–7. 10.1007/s00261-011-9737-6. 21512724 10.1007/s00261-011-9737-6

[3] Fattahi R, Balci NC, Perman WH, Hsueh EC, Alkaade S, Havlioglu N, et al. Pancreatic diffusion-weighted imaging (DWI): comparison between mass-forming focal pancreatitis (FP), pancreatic cancer (PC), and normal pancreas. J Magn Reson Imaging. 2009;29:350–6. 10.1002/jmri.21651. 19161187 10.1002/jmri.21651

[4] Achiam MP, Løgager VB, Skjoldbye B, Møller JM, Lorenzen T, Rasmussen VL, et al. Preoperative CT versus diffusion weighted magnetic resonance imaging of the liver in patients with rectal cancer; a prospective randomized trial. PeerJ. 2016;4:e1532. 10.7717/peerj.1532. 26793420 10.7717/peerj.1532 PMC4715462

[5] Macera A, Lario C, Petracchini M, Gallo T, Regge D, Floriani I, et al. Staging of colorectal liver metastases after preoperative chemotherapy. Diffusion-weighted imaging in combination with Gd-EOB-DTPA MRI sequences increases sensitivity and diagnostic accuracy. Eur Radiol. 2013;23:739–47. 10.1007/s00330-012-2658-0. 22976920 10.1007/s00330-012-2658-0

[6] Agarwal S, Grajo JR, Fuentes-Orrego JM, Abtahi SM, Harisinghani MG, Saini S, et al. Distinguishing hemangiomas from metastases on liver MRI performed with gadoxetate disodium: value of the extended washout sign. Eur J Radiol. 2016;85:635–40. 10.1016/j.ejrad.2015.12.028. 26860678 10.1016/j.ejrad.2015.12.028

[7] Litjens G, Riviere DM, van Geenen EJM, Radema SA, Brosens LAA, Prokop M, et al. Diagnostic accuracy of contrast-enhanced diffusion-weighted MRI for liver metastases of pancreatic cancer: towards adequate staging and follow-up of pancreatic cancer - DIA-PANC study: study protocol for an international, multicenter, diagnostic trial. BMC Cancer. 2020;20:744. 10.1186/s12885-020-07226-0. 32778061 10.1186/s12885-020-07226-0 PMC7418197

[8] Wang F, Dong Z, Reese TG, et al. Echo planar time-resolved imaging (EPTI). Magn Reson Med. 2019;81:3599–615. 30714198 10.1002/mrm.27673 PMC6435385

[9] Morani AC, Elsayes KM, Liu PS, et al. Abdominal applications of diffusion-weighted magnetic resonance imaging: Where do we stand. World J Radiol. 2013;5:68–80. 23671743 10.4329/wjr.v5.i3.68 PMC3650207

[10] Holland D, Kuperman JM, Dale AM. Efficient correction of inhomogeneous static magnetic field-induced distortion in echo planar imaging. Neuroimage. 2010;50:175–83. 19944768 10.1016/j.neuroimage.2009.11.044 PMC2819607

[11] Bammer R, Keeling SL, Augustin M, Pruessmann KP, Wolf R, Stollberger R, et al. Improved diffusion-weighted single-shot echo-planar imaging (EPI) in stroke using sensitivity encoding (SENSE). Magn Reson Med. 2001;46:548–54. 10.1002/mrm.1226. 11550248 10.1002/mrm.1226

[12] Yanasak NE, Kelly MJ. MR imaging artifacts and parallel imaging techniques with calibration scanning: a new twist on old problems. Radiographics. 2014;34:532–48. 10.1148/rg.342135051. 24617696 10.1148/rg.342135051

[13] Lustig M, Donoho D, Pauly JM. Sparse MRI: the application of compressed sensing for rapid MR imaging. Magn Reson Med. 2007;58:1182–95. 10.1002/mrm.21391. 17969013 10.1002/mrm.21391

[14] Nagata S, Goshima S, Noda Y, Kawai N, Kajita K, Kawada H, et al. Magnetic resonance cholangiopancreatography using optimized integrated combination with parallel imaging and compressed sensing technique. Abdom Radiol. 2019;44:1766–72. 10.1007/s00261-018-01886-0. 10.1007/s00261-018-01886-0 30659308

[15] Bratke G, Rau R, Weiss K, Kabbasch C, Sircar K, Morelli JN, et al. Accelerated MRI of the lumbar spine using compressed sensing: quality and efficiency. J Magn Reson Imaging. 2019;49:e164–75. 10.1002/jmri.26526. 30267462 10.1002/jmri.26526

[16] de Jonge CS, Coolen BF, Peper ES, Motaal AG, Nio CY, Somers I, et al. Evaluation of compressed sensing MRI for accelerated bowel motility imaging. Eur Radiol Exp. 2019;3:7. 10.1186/s41747-018-0079-9. 30725241 10.1186/s41747-018-0079-9 PMC6365583

[17] Kaga T, Noda Y, Mori T, Kawai N, Takano H, Kajita K, et al. Diffusion-weighted imaging of the abdomen using echo planar imaging with compressed SENSE: feasibility, image quality, and ADC value evaluation. Eur J Radiol. 2021;142:109889. 10.1016/j.ejrad.2021.109889. 34388627 10.1016/j.ejrad.2021.109889

[18] Kaga T, Noda Y, Asano M, Kawai N, Kajita K, Hyodo F, et al. Diagnostic ability of diffusion-weighted imaging using echo planar imaging with compressed SENSE (EPICS) for differentiating hepatic hemangioma and liver metastasis. Eur J Radiol. 2023;167:111059. 10.1016/j.ejrad.2023.111059. 37643558 10.1016/j.ejrad.2023.111059

[19] Laino ME, Vigano L, Ammirabile A, Lofino L, Generali E, Francone M, et al. The added value of artificial intelligence to LI-RADS categorization: a systematic review. Eur J Radiol. 2022;150:110251. 35303556 10.1016/j.ejrad.2022.110251

[20] Kelly BS, Judge C, Bollard SM, Clifford SM, Healy GM, Aziz A, et al. Radiology artificial intelligence: a systematic review and evaluation of methods (RAISE). Eur Radiol. 2022;32:7998–8007. 35420305 10.1007/s00330-022-08784-6 PMC9668941

[21] Lin DJ, Johnson PM, Knoll F, Lui YW. Artificial intelligence for MR image reconstruction: an overview for clinicians. J Magn Reson Imaging. 2021;53:1015–28. 32048372 10.1002/jmri.27078 PMC7423636

[22] Chaudhari AS, Sandino CM, Cole EK, Larson DB, Gold GE, Vasanawala SS, et al. Prospective deployment of deep learning in MRI: a framework for important considerations, challenges, and recommendations for best practices. J Magn Reson Imaging. 2021;54:357–71. 32830874 10.1002/jmri.27331 PMC8639049

[23] Pezzotti N, Yousefi S, Elmahdy MS, Van Gemert JHF, Schuelke C, Doneva M, et al. An adaptive intelligence algorithm for undersampled knee MRI reconstruction. IEEE Access. 2020;8:204825–38.

[24] Foreman SC, Neumann J, Han J, Harrasser N, Weiss K, Peeters JM, et al. Deep learning-based acceleration of compressed sense MR imaging of the ankle. Eur Radiol. 2022;32:8376–85. 35751695 10.1007/s00330-022-08919-9 PMC9705492

[25] Fujima N, Nakagawa J, Kameda H, Ikebe Y, Harada T, Shimizu Y, et al. Improvement of image quality in diffusion-weighted imaging with model-based deep learning reconstruction for evaluations of the head and neck. Magn Reson Mater Phys Biol Med. 2024;37:439–47. 10.1007/s10334-023-01129-4. 10.1007/s10334-023-01129-4 37989922

[26] Nishioka N, Fujima N, Tsuneta S, Yoshikawa M, Kimura R, Sakamoto K, et al. Enhancing the image quality of prostate diffusion-weighted imaging in patients with prostate cancer through model-based deep learning reconstruction. Eur J Radiol Open. 2024;13:100588. 39070063 10.1016/j.ejro.2024.100588 PMC11276920

[27] Klotz T, Montoriol PF, Da Ines D, Petitcolin V, Joubert-Zakeyh J, Garcier JM. Hepatic haemangioma: common and uncommon imaging features. Diagn Interv Imaging. 2013;94:849–59. 10.1016/j.diii.2013.04.008. 23796395 10.1016/j.diii.2013.04.008

[28] Ozaki K, Higuchi S, Kimura H, Gabata T. Liver metastases: correlation between imaging features and pathomolecular environments. Radiographics. 2022;42:1994–2013. 10.1148/rg.220056. 36149824 10.1148/rg.220056

[29] Heverhagen JT. Noise measurement and estimation in MR imaging experiments. Radiology. 2007;245:638–9. 18024445 10.1148/radiol.2453062151

[30] Xi Y, Liu A, Olumba F, Lawson P, Costa DN, Yuan Q, et al. Low-to-high b value DWI ratio approaches in multiparametric MRI of the prostate: feasibility, optimal combination of b values, and comparison with ADC maps for the visual presentation of prostate cancer. Quant Imaging Med Surg. 2018;8:557–67. 10.21037/qims.2018.06.08. 30140618 10.21037/qims.2018.06.08 PMC6081353

[31] Sullivan GM, Artino AR Jr. Analyzing and interpreting data from likert-type scales. J Grad Med Educ. 2013;5(4):541–2. 10.4300/JGME-5-4-18. 24454995 10.4300/JGME-5-4-18 PMC3886444

[32] Koo TK, Li MY. A guideline of selecting and reporting intraclass correlation coefficients for reliability research. J Chiropr Med. 2016;15:155–63. 27330520 10.1016/j.jcm.2016.02.012 PMC4913118

[33] Landis JR, Koch GG. The measurement of observer agreement for categorical data. Biometrics. 1977;33:159–74. 843571

[34] Kim DH, Kim B, Lee HS, Benkert T, Kim H, Choi JI, et al. Deep learning-accelerated liver diffusion-weighted imaging: intraindividual comparison and additional phantom study of free-breathing and respiratory-triggering acquisitions. Invest Radiol. 2023;58:782–90. 10.1097/RLI.0000000000000988. 37212468 10.1097/RLI.0000000000000988

[35] Bae SH, Hwang J, Hong SS, Lee EJ, Jeong J, Benkert T, et al. Clinical feasibility of accelerated diffusion weighted imaging of the abdomen with deep learning reconstruction: comparison with conventional diffusion weighted imaging. Eur J Radiol. 2022;154:110428. 10.1016/j.ejrad.2022.110428. 35797791 10.1016/j.ejrad.2022.110428

[36] Lemainque T, Yoneyama M, Morsch C, Iordanishvili E, Barabasch A, Schulze-Hagen M, et al. Reduction of ADC bias in diffusion MRI with deep learning-based acceleration: a phantom validation study at 3.0 T. Magn Reson Imaging. 2024;110:96–103. 10.1016/j.mri.2024.04.018. 38631532 10.1016/j.mri.2024.04.018

[37] Tokgoz O, Unlu E, Unal I, Serifoglu I, Oz I, Aktas E, et al. Diagnostic value of diffusion weighted MRI and ADC in differential diagnosis of cavernous hemangioma of the liver. Afr Health Sci. 2016;16:227–33. 10.4314/ahs.v16i1.30. 27358636 10.4314/ahs.v16i1.30 PMC4915395

[38] Inan N, Kilinc F, Sarisoy T, Gumustas S, Akansel G, Demirci A. Diffusion weighted MR imaging in the differential diagnosis of haemangiomas and metastases of the liver. Radiol Oncol. 2010;44:24–9. 10.2478/v10019-010-0001-4. 22933887 10.2478/v10019-010-0001-4 PMC3423674

[39] Lewis S, Dyvorne H, Cui Y, Taouli B. Diffusion-weighted imaging of the liver: techniques and applications. Magn Reson Imaging Clin N Am. 2014;22:373–95. 10.1016/j.mric.2014.04.009. 25086935 10.1016/j.mric.2014.04.009 PMC4121599

